# Aging of alveolar type 2 cells induced by *Lonp1* deficiency exacerbates pulmonary fibrosis

**DOI:** 10.17305/bb.2024.10429

**Published:** 2024-10-01

**Authors:** Weiwei Zhu, Chunting Tan, Jie Zhang

**Affiliations:** 1 Department of Pulmonary and Critical Care Medicine, Beijing Tiantan Hospital, Capital Medical University, Beijing, China; 2 Department of Pulmonary and Critical Care Medicine, Beijing Friendship Hospital, Capital Medical University, Beijing, China

**Keywords:** Idiopathic pulmonary fibrosis (IPF), lon protease 1 (*Lonp1*) gene, senescence.

## Abstract

Idiopathic pulmonary fibrosis (IPF) is a progressive and chronic disease that significantly impacts patient quality of life, and its incidence is on the rise. The pathogenesis of IPF remains poorly understood. Alveolar type 2 (AT2) cells are crucial in the onset and progression of IPF, yet the specific mechanisms involved are not well defined. Lon protease 1 (LONP1), known for its critical roles in various diseases, has an unclear function in IPF. Our research investigated the impact of *Lonp1* gene deletion on AT2 cell functionality and its subsequent effect on IPF development. We generated a bleomycin-induced pulmonary fibrosis mouse model with a targeted *Lonp1* knockout in AT2 cells and assessed the consequences on AT2 cell function and fibrosis progression. Additionally, we constructed the MLE12 cells with stable *Lonp1* knockdown and utilized transcriptome sequencing to identify pathways altered by the *Lonp1* knockdown. Our results indicated that mice with AT2 cell-specific *Lonp1* knockout exhibited more severe fibrosis compared to controls. These mice exhibited a reduction in AT2 and AT1 cell populations, along with an increase in p53- and p21-positive AT2 cells. *Lonp1* knockdown in MLE12 cells led to the upregulation of aging-associated pathways, with fibroblast growth factor 2 (*Fgf2)* gene emerging as a central gene interconnecting these pathways. Therefore, loss of *Lonp1* appears to promote AT2 cell aging and exacerbate bleomycin-induced pulmonary fibrosis. *Fgf2* emerges as a pivotal downstream gene associated with cellular senescence. This study uncovers the role of the *Lonp1* gene in pulmonary fibrosis, presenting a novel target for investigating the pathological mechanisms and potential therapeutic approaches for IPF.

## Introduction

Lon protease 1 (LONP1), encoded by the *LONP1* gene, is an ATPase located in the mitochondrial matrix that participates in the mitochondrial unfolded protein response. LONP1 specifically recognizes and binds multiple signals in unfolded peptides, distinguishing irreversibly damaged proteins from normal transiently unfolded proteins. Through these actions, LONP1 contributes to maintaining protein quality control and homeostasis in cells [1]. Additionally, LONP1 prevents the oxidation, aggregation, and accumulation of aconitase and glutamine, which are involved in the tricarboxylic acid cycle, thereby regulating mitochondrial energy metabolism [2]. Another study showed that LONP1 regulates mitochondrial DNA (mtDNA) replication and transcription by regulating the degradation of human mitochondrial transcription factor A (TFAM) [3]. LONP1 is also involved in mitochondrial autophagy through the regulation of eucaryotic initiation factor 2 alpha (eIF2α) [4].

Impaired protein quality regulation and mitochondrial dysfunction are common contributors to aging and age-related diseases [5]. LONP1 plays an important role in maintaining mitochondrial homeostasis and controlling protein quality [6–8]. Studies have shown that the downregulation of *Lonp1* expression or decreased activity of LONP1 promotes cellular aging and age-related diseases [9, 10]. The content of LONP1 in the liver tissue of elderly animals is lower than that of young animals [11]. Recent studies have shown that the expression of LONP1 is reduced in both aging cartilage and replicative aging chondrocytes. This may be related to changes in nuclear gene expression caused by aging.

Idiopathic pulmonary fibrosis (IPF) is a progressive, chronic disease that causes fibrosis of lung tissue. The prognosis of IPF is poor, and its incidence is on the rise. Although some treatments have been developed to delay the deterioration of lung function, curative treatments are still lacking. Alveolar type 2 (AT2) cells play important roles in secretion and regeneration in the alveoli and are involved in regulating inflammation and maintaining immune balance. Numerous studies have shown that AT2 cell dysfunction is one of the driving factors of pulmonary fibrosis. The widely accepted view is that the depletion of AT2 cells and the acquisition of aging-related secretory phenotypes are the fundamental mechanisms underlying the involvement of AT2 cells in IPF. One study demonstrated that the deficiency of the switch-independent 3a (Sin3a) regulator initiated p53-dependent cellular aging in AT2 cells, thus promoting the occurrence and development of IPF. This finding suggested that the key determinant of IPF occurrence is AT2 cell aging, rather than AT2 cell depletion [12]. Previous studies have shown that senolytics, drugs specifically targeting aging cells, can alleviate pulmonary fibrosis in mice by depleting aging AT2 cells [13]. Clinical trials have also shown that senolytics can improve the physical function of IPF patients [14, 15].

In this study, we examined the potential function of *Lonp1* in IPF by establishing a mice model with AT2 cell-specific knockout of *Lonp1* and inducing experimental pulmonary fibrosis using bleomycin. We further evaluated whether the loss of *Lonp1* induces aging in AT2 cells and contributes to the development of IPF.

## Materials and methods

### Animals

Surfactant protein C (*Sftpc*)^CreERT2^ mice were acquired from The Jackson Laboratory, and *Lonp1*^flox/flox^ mice were purchased from Jicui Yaokang Biotechnology Co., Ltd. We used mice aged 12–16 weeks for the experiments. The mice were housed in a room maintained at constant temperature and humidity, with 12-h light/dark cycles, and had free access to standard food and tap water.

To generate the *Sftpc*^CreERT2^; *Lonp1*^flox/+^ mice, *Sftpc*^CreERT^ male mice were bred with *Lonp1*^flox/flox^ female mice. To generate the *Sftpc*^CreERT2^; *Lonp1*^flox/flox^ mice, we bred the *Sftpc*^CreERT2^; *Lonp1*^flox/+^male mice with the *Lonp1*^flox/flox^ female mice. Mice of genotype *Sftpc*^CreERT2^; *Lonp1*^flox/+^ from the same litter served as the control group. To construct AT2 cell-specific *Lonp1* knockout mice, tamoxifen (Sigma Aldrich, CAS No. 10540-29-1), dissolved in corn oil, was administered intraperitoneally at a dose of 75 mg/kg daily for five days.

To confirm the mouse genotypes, DNA was extracted from tails and analyzed using the polymerase chain reaction (PCR). The primer sequences were as follows: Cre-F: 5′-CGTACTGACGGGAAT-3′, Cre-R: 5′ TGCATGATCCCGGTATTGA-3′; Flox-F: 5′-GCAGAGAAGCATGAGTGCA-3′, and Flox-R: 5′-TAAGTAGTCCAGGGGTTAG-3′. The PCR products were analyzed by gel electrophoresis and the genotypes were confirmed by the following band sizes: Cre, 400 bp; Flox-*Lonp1*, 391 bp; and wild type (WT), 286 bp.

### Bleomycin-induced fibrosis model

On the 7th day after the final administration of tamoxifen, a pulmonary fibrosis model was constructed in mice by bleomycin induction. The mice were anesthetized with inhaled isoflurane. AT2 cell-specific *Lonp1* knockout groups (aged 12–16 weeks, weighting 22–34 g; five mice per group) were administered 2 mg/kg bleomycin (15 mg sourced from Nippon Chemical Pharmaceutical Co., Ltd.) via tracheal intubation in a volume of 50 µL. The control groups received an equal volume of physiological saline through tracheal intubation. The weight and health status of all mice were closely monitored, with any mouse experiencing a weight loss of 20% or more being euthanized. Two weeks after the tracheal instillation of either bleomycin or saline, all mice were euthanized, and their lung tissues were harvested for further analysis.

### Preparation of mouse serum samples

After anesthetization, the mice’s whiskers were trimmed to prevent hemolysis, which could occur if blood contacted the whiskers. To facilitate blood collection, the skin around one side of the mouse’s eye was gently squeezed to slightly protrude the eyeball. Using alcohol-disinfected, pointed curved forceps, the eyeball was carefully clamped and quickly removed to collect blood in a centrifuge tube. The collected blood was then allowed to stand at room temperature for 1 h before being centrifuged at 3000 rpm and 4 ^∘^C for 10 min. The resulting serum sample was stored at −80 ^∘^C for further analysis.

### Histopathological analysis

The left lungs of the mice were fixed in 4% paraformaldehyde and subsequently embedded in paraffin. The tissue blocks were then sectioned into 4-µm slices. These slices were stained with hematoxylin and eosin (HE) using standard procedures. Additionally, Masson’s staining was performed with a Masson’s Trichrome Stain Kit (G1346, Solarbio, China), following the manufacturer’s instructions. The severity of pulmonary fibrosis was evaluated using an upright optical microscope (CX23, Leica, Germany) at ×100 magnification. The extent of fibrosis was quantified using the Ashcroft score [16]. For each tissue slice, at least five fields of view were examined to calculate an average score, which was used to evaluate the degree of pulmonary fibrosis in the mouse lung tissue.

### Immunofluorescence staining

Paraffin sections were subjected to dewaxing and hydration, antigen repair, and blocking with goat serum. The sections were then incubated overnight at 4 ^∘^C with primary antibodies: collagen III (ab7778, 1:200, Abcam), anti-prosurfactant protein C (AB3786, 1:200, Sigma Aldrich), HOP homeobox (Hopx) (11419-1-AP, 1:200, Proteintech), and alpha-smooth muscle actin (α-SMA) (GB13044-50, 1:200, Servicebio). This was followed by a 1-h room temperature incubation with secondary antibodies: Alexa fluor 488-labeled goat anti-rabbit IgG (GB25303, Servicebio) and Alexa fluor 594–labeled goat anti-rabbit IgG (GB28301, Servicebio). The samples were then sealed with anti-fluorescence quencher (P36962, Invitrogen) and stained with 4′,6-diamidino-2-phenylindole (DAPI). The sections were examined using a confocal laser scanning microscope (FV10, Olympus, Tokyo, Japan) under ×200 magnification. At least five fields of view were analyzed for each slice. Quantification of staining was accomplished using ImageJ software.

### Immunohistochemistry

Paraffin sections were subjected to dewaxing and hydration, antigen repair, and peroxidase inhibitor treatment for 10 min. After blocking with goat serum, the sections were incubated overnight at 4 ^∘^C with primary antibodies: anti-prosurfactant protein C (AB3786, 1:200, Abcam), p53 (sc-126, 1:200, Santa Cruz Biotechnology), and p21 (ab188224, 1:200, Abcam). This was followed by a 30-min incubation with a horseradish peroxidase (HRP)-conjugated secondary antibody. The sections were then stained with DAB (diaminobenzidine) and counterstained with hematoxylin before being sealed with neutral resin. The stained sections were examined using an upright optical microscope (CX23, Leica, Germany) at ×100 magnification. At least five fields of view were analyzed for each slice. Quantification of staining was performed using ImageJ software.

### Western blot

Mouse lung tissue was homogenized in precooled radioimmunoprecipitation assay (RIPA) lysis buffer (Strong) (HY-K1001, MCE, China). The lysate was then centrifuged at 12000 rpm for 30 min at 4 ^∘^C, and the supernatant was collected. The total protein concentration in the supernatant was determined using the bicinchoninic acid (BCA) method (P0010, Beyotime, China). Proteins were separated using a 10% sodium dodecyl sulfate-polyacrylamide gel electrophoresis (SDS-PAGE) and subsequently transferred to polyvinylidene difluoride (PVDF) membranes. These membranes were blocked with 5% skimmed milk at room temperature for 2 h, followed by overnight incubation at 4 ^∘^C with primary antibodies: transforming growth factor beta 1 (TGFβ-1) (ab21571, 1:1000, Abcam), glyceraldehyde 3-phosphate dehydrogenase (GAPDH) (60,004-1-lg, 1:10,000, Proteintech), *Lonp*1 (66043-1-Ig, 1:2000, Proteintech), and α-tubulin (ab40742, 1:5000, Abcam). The membranes where washed with tris-buffered saline with tween (TBST), followed by a 1.5-h incubation at room temperature with secondary antibodies: goat anti-rabbit (SA00001-2, 1:5000, Proteintech) and goat anti-mouse (SA00001-1, 1:5000, Proteintech). Protein bands were visualized using the Ultra High Sensitivity ECL Kit (HY-K1005, Med Chem Express, NJ, USA) and imaged with a system from Bio-Rad (Hercules, CA, USA). The bands were quantified using ImageJ software.

### Enzyme-linked immunosorbent assay (ELISA)

Mouse blood was centrifuged at 4000 rpm for 10 min, and the serum was collected. The concentration of plasminogen activator inhibitor-1 (PAI-1) was measured using an ELISA kit (BQEM-556-96T, Biosharp) following the manufacturer’s instructions. The optical density (OD) was measured at 450 nm.

### Cell line and cell culture

MLE12 cells were obtained from iCell (iCell-m036). These cells were cultured in MLE12-specific medium (iCell-m036-001b) in a 37 ^∘^C incubator with 5% CO_2_. MLE12 cells were purchased from iCell (iCell-m036).

### Small interfering RNA (siRNA) transfection

Twenty-four hours before transfection, MLE12 cells were seeded in a 6-well plate at a density of 1.5 × 10^5^ cells per well. The siRNAs targeting *Lonp1* and the control siRNAs were procured from Genomeditech. For the transfection, siRNA was diluted in 250 µL of the optimized minimal essential medium (Opti-MEM) to a final concentration of 25 nM. Separately, 7.5 µL of lipofectamine RNAiMAX (13778150, ThermoFisher, USA) was mixed with 250-µL Opti-MEM. After allowing both solutions to rest at room temperature for 5 min, they were combined and held for an additional 10 min at room temperature. The transfection complex was then added in a 6-well cell plate, and the cells were cultured in a 37 ^∘^C incubator with 5% CO_2_. The culture medium was replaced 12-h post-transfection.

### Cell cycle analysis

MLE12 cells transfected with siRNA were treated with either phosphate-buffered saline (PBS) or bleomycin (5 µg/mL) for 72 h. Post-treatment, cells were harvested and analyzed using a cell cycle analysis kit (Beyotime, C1052). Red fluorescence was detected using a flow cytometer at an excitation wavelength of 488 nm. Data were analyzed using FlowJo software.

### Lentivirus

The lentivirus was produced using a three-plasmid system. This system included shuttle vectors for *Lonp1* gene interference and two auxiliary plasmids, H1 (expressing group-specific antigen [*gag*]/polymerase [pol] and regulator of expression of virion proteins [rev]) and H2 (expressing the cell membrane protein vesicular stomatitis virus glycoprotein [VSVG]). These plasmids were co-transfected into 293T cells. The virus-containing supernatant was collected and used to infect MLE12 cells. After 12 h of infection, the medium was replaced with fresh culture medium. After 72 h, puromycin was added to a final concentration of 1.5 µg/mL. The medium containing puromycin was refreshed every 2–3 days.

### Transcriptome sequencing

MLE-12 cells stably expressing lentivirus-mediated short hairpin RNA (shRNA) targeting *Lonp1*, along with control cells, were harvested using the total RNA isolation (TRI) reagent (Sigma-Aldrich, T9424). The samples were subsequently stored at −80 ^∘^C. These cell samples were sent to Gene Denovo Biotechnology Co., Ltd. (Guangzhou, China) for transcriptome sequencing.

### Differential gene clustering heatmap

Differential expression analysis was conducted on the gene expression levels of each sample, which were processed by converting to log10 of fragments per kilobase of transcript per million mapped reads (FPKM) or log10 of transcripts per million (TPM). Hierarchical clustering was performed on differentially expressed genes. The results were visualized using heatmaps. Genes were considered significantly upregulated or downregulated if they exhibited a fold change > 1.5 with a *P* value < 0.05.

### Gene set enrichment analysis (GSEA)

We conducted a GSEA using the GSEA software and the Molecular Signatures Database (MSigDB) to evaluate whether a set of genes in specific Kyoto Encyclopedia of Genes and Genomes (KEGG) pathways exhibited significant differences between two groups. The Signal2Noise metric was used to sort the genes. We calculated the enrichment score (ES), *P* value, and the false discovery rate (FDR) using the default parameters. Gene sets were considered significant if they had a normalized ES (NES) with an absolute value greater than 1, a nominal *P* value (NOM *P* val) less than 0.05, and an FDR less than 0.25. A larger absolute NES value and a smaller FDR indicate higher credibility of the analysis results.

### Protein interaction network analysis

We performed the protein interaction network analysis using the Search Tool for the Retrieval of Interacting Genes/Proteins (STRING) protein interaction database (http://string-db.org) [17]. For species represented in the database, we extracted a set of differentially expressed genes and used Cytoscape [18] to construct an interaction network diagram. For species not included in the database, we first applied the Basic Local Alignment Search Tool x (BLASTx) alignment of the protein sequences from our target gene set against the protein sequences of reference species in the STRING database. We then constructed an interaction network diagram based on the protein interaction relationships of the reference species from the alignment results.

### Quantitative reverse transcription (RT)-PCR analysis

The lung lobes (20 mg) were homogenized in 0.5 mL of Trizol reagent (Sigma-Aldrich, T9424). The complementary DNA (cDNA) was synthesized using PrimeScript RT Master Mix (Takara, RR036A) following the manufacturer’s instructions. Real-time RT-PCR was conducted using Hieff qPCR SYBR Green Master Mix (Yeasen, 11199ES03) on a 7500Fast real-time PCR detection system (Thermo Fisher). The specific primer sequences were as follows: fibroblast growth factor 2 (*Fgf2)*-F: 5′-GCGACCCACACGTCAAACTA-3′ and *Fgf2*-R: 5′-CCGTCCATCTTCCTTCATAGC-3′. The thermal cycling conditions included an initial denaturation at 95 ^∘^C for 5 min, followed by 40 cycles of 95 ^∘^C for 10 s, 60 ^∘^C for 20 s, and 72 ^∘^C for 40 s. Relative gene expression levels were quantified using the comparative cycle threshold (CT) method.

### Gene Expression Omnibus (GEO) database analysis

Data were sourced from the GSE70866 dataset in the GEO database (https://www.ncbi.nlm.nih.gov/geo) [19]. The raw data were obtained in the MINiML file format. For visualization, box plots of the data were generated using the “boxplot” function.

### Ethical statement

All animal studies were approved by the Animal Care and Use Committee of Beijing Friendship Hospital, Capital Medical University, Beijing, China (approval number 21-2001).

### Statistical analysis

Statistical analyses were performed using GraphPad Prism 9 (GraphPad Software, Inc., San Diego, CA, USA). Comparisons between two groups were conducted using a *t*-test, while analysis of variance (ANOVA) was used for comparisons involving more than two groups. A *P* < 0.05 indicated a statistically significant difference.

## Results

### Knockout of *Lonp1* in AT2 cells exacerbates bleomycin-induced pulmonary fibrosis in mice

**Figure 1.**
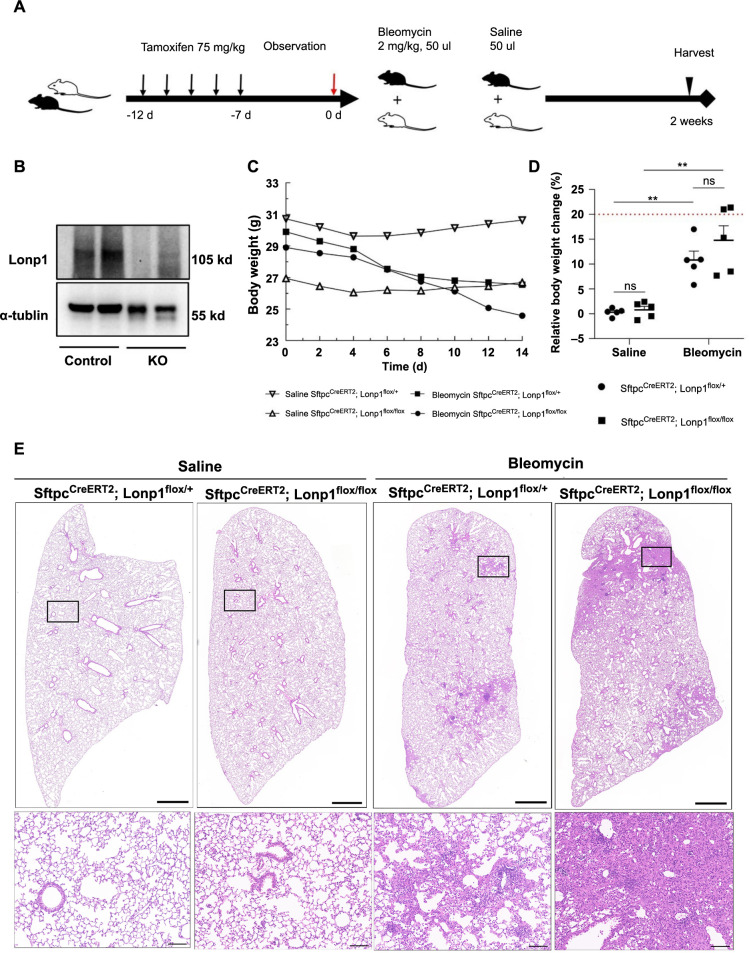
***Lonp1* gene knockout exacerbates bleomycin-induced lung structural damage.** (A) Depicting the experimental protocol schematic. Black mice represent *Sftpc*^CreERT2^*; Lonp1*^flox/flox^ mice, while the white mice represent *Sftpc*^CreERT2^*; Lonp1*^flox/+^ mice. The black arrows indicate days of tamoxifen injection, and the red arrow denotes the administration of bleomycin or saline (*n* ═ 5 per group); (B) Displaying the validation of *Lonp1* gene knockout in AT2 cells. “Control” denotes *Sftpc*^CreERT2^*; Lonp1*^flox/+^ mice, and “KO” denotes *Sftpc*^CreERT2^*; Lonp1*^flox/flox^ mice; (C) A line graph plotting the body weight changes in mice over time after tracheal instillation of bleomycin or saline; (D) A graph depicting the percentage of weight change in mice before and after treatment with bleomycin or saline. The red dashed line marks a 20% weight loss threshold in the mice; (E) HE staining of lung tissue sections from the indicated groups of mice. Scale bars: top panels ═ 1000 µm; bottom panels ═ 100 µm. *P < 0.05; **P < 0.01; ***P < 0.001; ****P < 0.0001. *Lonp1*: Lon protease 1; *Sftpc*: Surfactant protein C; AT2 cells: Alveolar type 2 cells; HE: Hematoxylin and eosin.

To investigate the role of *Lonp1* in pulmonary fibrosis, we specifically knocked out the *Lonp1* gene in AT2 cells by generating *Sftpc*^CreERT2^*; Lonp1*^flox/flox^ mice, as detailed in the Methods section. These mice were intraperitoneally injected with tamoxifen (75 mg/kg) over five days to achieve *Lonp1* knockout in AT2 cells. Subsequently, to establish the bleomycin-induced pulmonary fibrosis mouse model, both the AT2 cell-specific *Lonp1* knockout mice group (*Sftpc*^CreERT2^*; Lonp1*^flox/flox^) and the littermate control group (*Sftpc*^CreERT2^*; Lonp1*^flox/+^) were treated with a tracheal instillation of bleomycin or physiological saline. The mice were euthanized two weeks later for lung tissue analysis, with the experimental protocol outlined in Figure 1A.

Post-tamoxifen treatment, *Lonp1* expression was virtually undetectable in the AT2 cell-specific *Lonp1* knockout group (Figure 1B). Both the knockout group and the control group experienced mild weight loss during the first four days post-treatment but gradually regained weight back to their initial weight (Figure 1C). However, following the administration of bleomycin, both groups showed continued weight loss. After six days, the AT2 cell-specific *Lonp1* knockout group mice exhibited a greater weight loss compared with the control group. Despite this, the differences in weight loss between the AT2 cell-specific *Lonp1* knockout group and the control group, regardless of bleomycin or saline treatment, were not statistically significant (Figure 1D).

**Figure 2.**
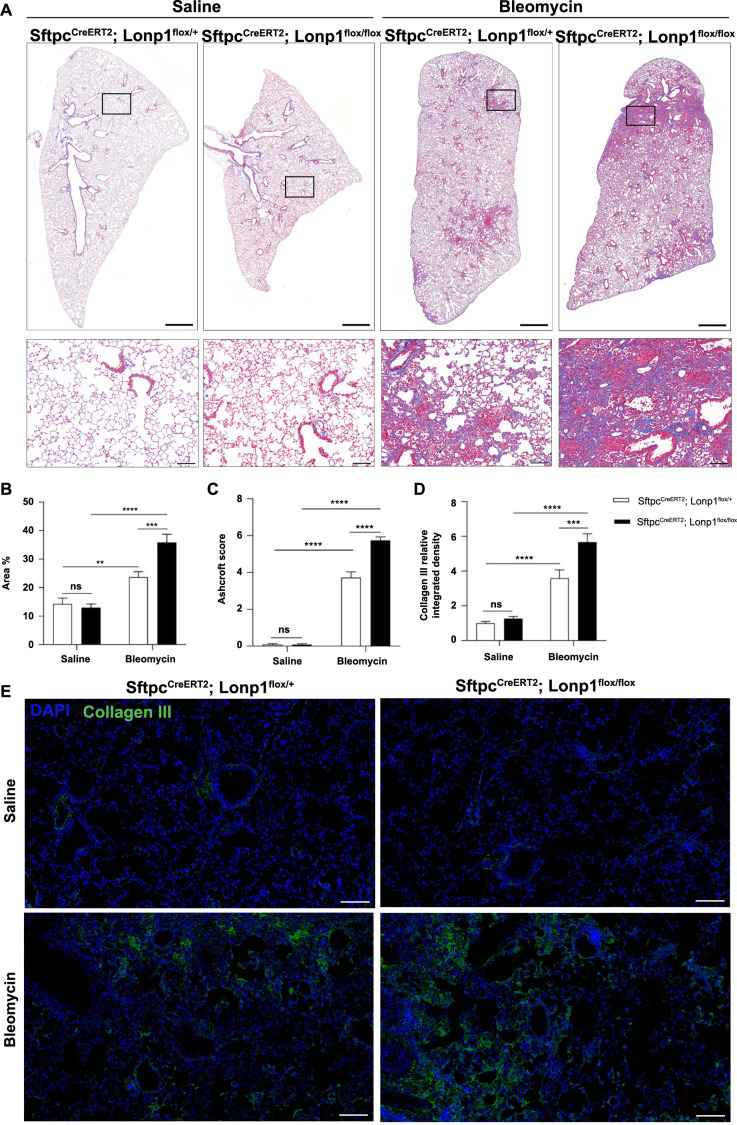
***Lonp1* gene knockout exacerbates bleomycin-induced pulmonary collagen deposition.** (A) Masson’s staining of lung tissue from the indicated mouse groups. Scale bars: top panels ═ 1000 µm; bottom panels ═ 100 µm; (B) Bar graph displaying the quantification analysis of collagen deposition areas as a percentage (Masson’s staining); (C) Bar graph illustrating the Ashcroft scoring of the indicated mouse groups; (D) Bar graph showcasing the relative quantification of integrated density values for collagen III (immunofluorescence staining); (E) Immunofluorescence staining for collagen III in lung tissue sections from the indicated mouse groups. Scale bar ═ 100 µm. *P < 0.05; **P < 0.01; ***P < 0.001; ****P < 0.0001. *Lonp1*: Lon protease 1; *Sftpc*: Surfactant protein C; DAPI: 4’,6-diamidino-2-phenylindole.

**Figure 3.**
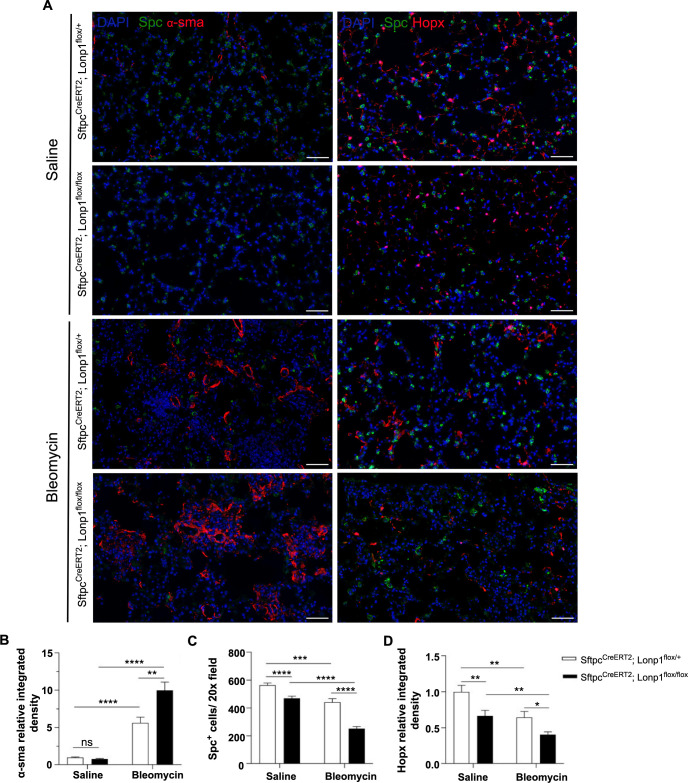
***Lonp1* gene knockout reduces the number of AT2 and AT1 cells.** (A) Immunofluorescence staining showcasing Spc^+^ cells, Hopx^+^ cells, and α-SMA in lung tissues from mice. Scale bar ═ 100 µm; (B) Bar graph displaying the relative quantification of relative integrated density values for α-SMA; (C) Bar graph depicting the quantitative analysis of the number of Spc^+^ cells; (D) Bar graph depicting the quantitative analysis of the Hopx^+^ cell count. *P < 0.05; **P < 0.01; ***P < 0.001; ****P < 0.0001. *Lonp1*: Lon protease 1; AT2 cells: Alveolar type 2 cells; AT1 cells: Alveolar type 1 cells; Spc: Surfactant rotein C; Hopx: HOP homeobox; α-SMA: Alpha-smooth muscle actin; *Sftpc*: Surfactant protein C; DAPI: 4’,6-diamidino-2-phenylindole.

HE staining of lung sections revealed that the AT2 cell-specific *Lonp1* knockout mice exhibited more extensive alveolar structural damage and increased fibrotic tissue masses following tracheal instillation of bleomycin (Figure 1E). Masson’s staining further confirmed that collagen deposition and fibrosis were more pronounced in the lungs of the AT2 cell-specific *Lonp1* knockout mice after bleomycin treatment (Figure 2A and 2B). This was supported by higher Ashcroft scores in the knockout group compared to the control group under bleomycin-treated conditions (Figure 2C). Immunofluorescence showed increased collagen III and α-SMA deposit levels in the AT2 cell-specific *Lonp1* knockout mice compared with the control mice after tracheal instillation of bleomycin (Figures 2D and 2E, 3A and 3B). Collectively, these results indicated that the loss of *Lonp1* leads to more severe pulmonary fibrosis.

### Knockout of *Lonp1* in AT2 cells reduces the number of AT2 and AT1 cells

We further examined the effects of *Lonp1* knockout on alveolar cell populations, utilizing immunohistochemical staining of the mouse lung tissue to identify AT1 cells with the marker Hopx and AT2 cells with the marker surfactant protein C (Spc). The expressions of Spc and Hopx were reduced in the lung tissue of the AT2 cell-specific *Lonp1* knockout mice compared with the control group, under both the bleomycin-treated and control conditions (Figure 3A). Notably, the decrease in Spc and Hopx expression in the lung tissue of mice in the AT2 cell-specific *Lonp1* knockout group was more significant in the bleomycin-treated groups (Figure 3C and 3D).

These results showed that knocking out *Lonp1* resulted in a decreased number of AT2 cells, as well as AT1 cells. Since mature AT2 cells serve as progenitors for AT1 cells postnatally [20], the observed decline in AT1 cells could be attributed to the compromised proliferative capacity of AT2 cells following the deletion of *Lonp1*.

### Knockout of *Lonp1* in AT2 cells promotes aging of AT2 cells

**Figure 4.**
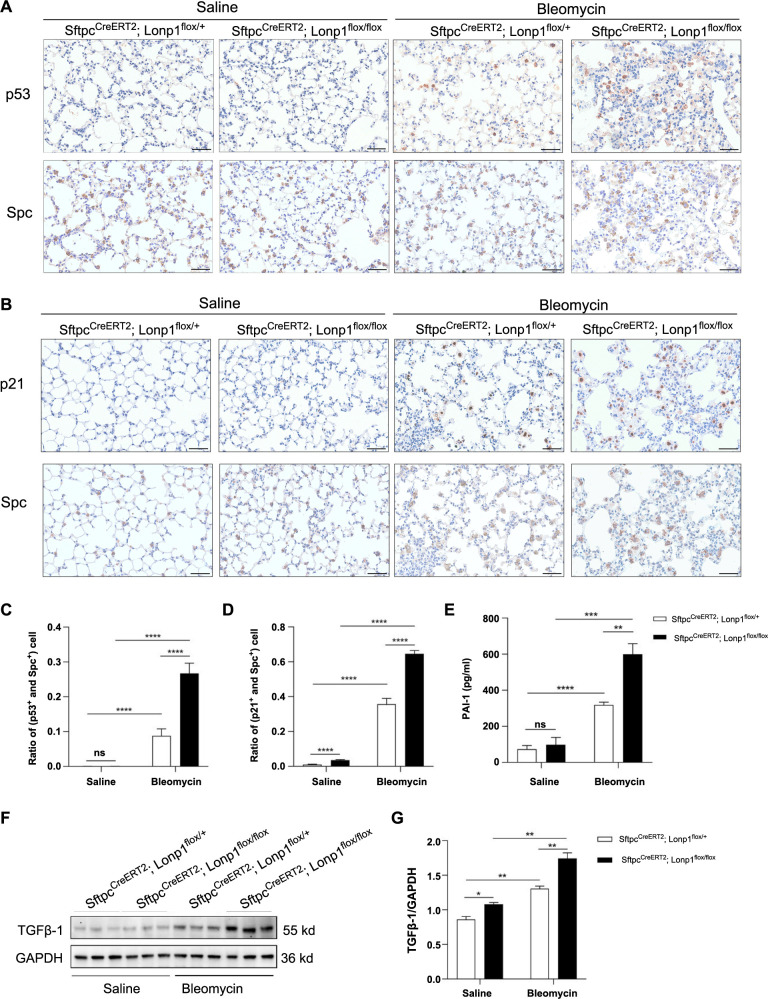
***Lonp1* gene knockout promotes bleomycin-induced aging of AT2 cells.** (A) Immunohistochemistry on consecutive lung tissue sections demonstrating the colocalization of p53 with Spc. Scale bar ═ 100 µm; (B) Immunohistochemistry on consecutive lung tissue sections demonstrating the colocalization of p21 with Spc. Scale bar ═ 100 µm; (C) Bar graph displaying the proportion of p53^+^and Spc^+^ double positive cells to Spc^+^ single positive cells; (D) Bar graph displaying the proportion of p21^+^and Spc^+^ double positive cells to Spc^+^ single positive cells; (E) Bar graph showcasing the concentration of PAI-1 in mouse serum; (F) Western blot analysis detecting the TGFβ-1 in mouse lung tissue; (G) Bar graph displaying the quantitative analysis of TGFβ-1 expression. *P < 0.05; **P < 0.01; ***P < 0.001; ****P < 0.0001. *Lonp1*: Lon protease 1; AT2 cells: Alveolar type 2 cells; Spc: Surfactant rotein C; PAI-1: Plasminogen activator inhibitor-1; TGFβ-1: Transforming growth factor beta 1; *Sftpc*: Surfactant protein C; GAPDH: Glyceraldehyde 3-phosphate dehydrogenase.

To investigate whether *Lonp1* affects AT2 cell numbers through cellular senescence, we assessed the expression of the aging markers p53 and p21 in AT2 cells within the lung tissues of mice [21]. Immunohistochemical analysis of consecutive sections confirmed the colocalization of p53 and p21 with Spc. In the AT2 cell-specific *Lonp1* knockout mice, bleomycin treatment resulted in an increased number of AT2 cells positive for p53 or p21 compared with the control group (Figure 4A–4D). Furthermore, to evaluate whether the knocking out of *Lonp1* affects the senescence-associated secretory phenotype (SASP), we measured the levels of two SASP-related secretory factors, PAI-1 in serum and TGFβ-1 in lung tissue. The AT2 cell-specific *Lonp1* knockout mice treated with bleomycin exhibited higher concentrations of PAI-1 and TGFβ-1 compared to bleomycin-treated control mice (Figure 4E–4G).

**Figure 5.**
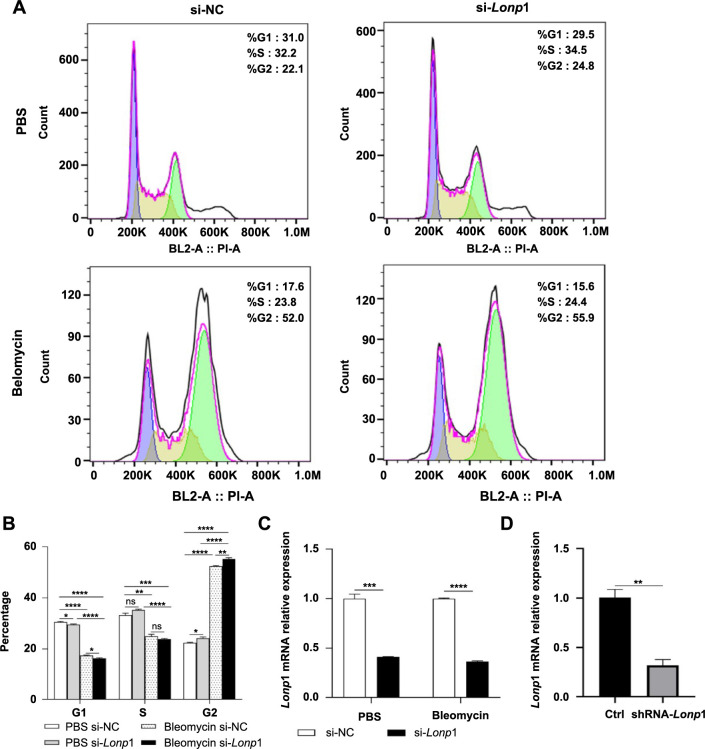
**Analysis of cell cycle alterations and *Lonp1* knockdown efficiency in MLE12 cells.** (A) Showcasing the flow cytometry analysis of the cell cycle profiles of MLE12 cells transfected with siRNAs, following treatment with PBS or bleomycin; (B) Bar graph representation of the percentage of cells in G1, S, and G2 phases of the cell cycle; (C) Bar graph showing relative mRNA expression levels confirming the knockdown efficiency of *Lonp1* in MLE12 cells treated with si-*Lonp1* compared to si-NC, following PBS and bleomycin treatment; (D) Bar graph showing relative mRNA expression levels confirming the knockdown efficiency of *Lonp1* post-lentiviral transduction of MLE12 cells with si-*Lonp1. Lonp1*: Lon protease 1; siRNA: Small interfering RNA; PBS: Phosphate-buffered saline; mRNA: Messenger RNA; NC: Negative control; BL2-A: Blue laser channel 2 area; PI-A: Propidium iodide area.

We further performed in vitro experiments to assess the effects of *Lonp1* knockdown on the cell cycle of MLE12 cells. Cells transfected with siRNA targeting *Lonp1* (si-*Lonp1*) or a non-targeting negative control (si-NC) were treated with PBS or bleomycin for 72 h, after which cell cycle distribution was analyzed. The results revealed that *Lonp1* knockdown exacerbated the G2 phase cell cycle arrest; the si-*Lonp1* group displayed an increased percentage of cells in the G2 phase and a decreased percentage in the G1 phase compared to the si-NC group, following both PBS and bleomycin treatment. Compared to the PBS-treated si-NC group, there was an elevation in the G2 phase percentage and a reduction in the G1 phase percentage in the bleomycin-treated si-NC group. A similar pattern was observed when comparing the PBS-treated si-*Lonp1* group to the bleomycin-treated si-*Lonp1* group (Figure 5A–5C). These observations suggested that bleomycin alone can induce cell cycle arrest, while the downregulation of the *Lonp1* gene further exacerbates the cell arrest. These results indicated that silencing of *Lonp1* promotes AT2 cell aging, enhances the expression of SASP, and exacerbates bleomycin-induced cell cycle arrest.

### Exploration of the pathways regulated by *Lonp1* by transcriptome sequencing

**Figure 6.**
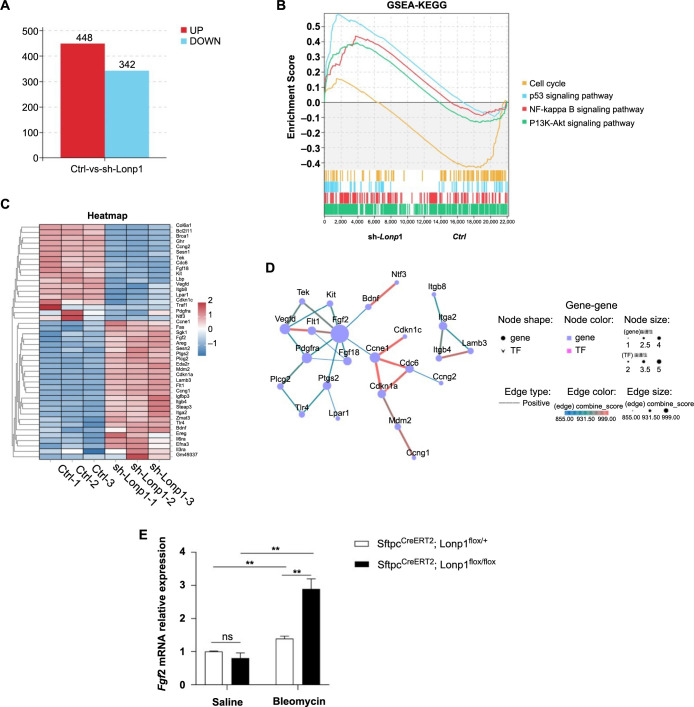
**The aging-related signaling pathways influenced by *Lonp1* gene knockout.** (A) Bar graph depicting the number of differentially expressed genes between the Ctrl and sh-*Lonp1* groups; (B and C) Volcano plot (B) illustrating the the top five upregulated and downregulated genes among the differentially expressed genes. A heatmap (C) and the GSEA-KEGG enrichment pathway analysis (B) highlighting the four aging-related pathways. (D) PPI network diagram illustrating the connections among differentially expressed genes within the four enriched pathways. Gene–gene interactions are shown, with node shapes indicating genes and transcription factors. (E) Bar graph showing the relative mRNA expression levels of the *Fgf2* gene in mouse lung tissue post-treatment with saline or bleomycin, validating its upregulation. (F) Analysis of *Lonp1* gene expression in the lung tissue of IPF patients compared to healthy controls. *Lonp1*: Lon protease 1; Ctrl: Control; shRNA: Short hairpin RNA; GSEA: Gene set enrichment analysis; KEGG: Kyoto Encyclopedia of Genes and Genomes; PPI: Protein–protein interaction; mRNA: Messenger RNA; *Fgf2*: Fibroblast growth factor 2; IPF: Idiopathic pulmonary fibrosis; UP: Upregulated; DOWN: Downregulated; PI3K-AKT: Phosphoinositide 3-kinase-protein kinase B; NF-kB: Nuclear factor kappa-light-chain-enhancer of activated B cells; *Sftpc*: Surfactant protein C; TF: Transcription factors.

To explore the underlying mechanisms of *Lonp1*, we generated MLE12 lung epithelial cells with stable knockdown of the *Lonp1* gene using lentiviral vectors (Figure 5D). We conducted transcriptome sequencing on both the control group (Ctrl) and the knockdown group (sh-*Lonp1*), uncovering 790 differentially expressed genes: 448 were upregulated, while 342 were downregulated (Figure 6A). A volcano plot highlighted the top five upregulated and downregulated genes among these differentially expressed genes (Figure 6B). Further analysis using GSEA identified four aging-related pathways affected by the differential gene expression: one downregulated pathway related to the cell cycle, and three upregulated pathways, specifically the p53 signaling, the phosphoinositide 3-kinase-protein kinase B (PI3K-AKT) signaling pathway, and the nuclear factor kappa-light-chain-enhancer of activated B cells (NF-kB) signaling pathway (Figure 6C). Notably, cyclin-dependent kinase inhibitor 1A *(Cdkn1a)*, the gene encoding p21, was identified as a differentially expressed gene.

We extended our investigation to a protein interaction analysis of the differentially expressed genes within the four aging-related pathways, which revealed that the *Fgf2* gene connected these pathways (Figure 6D). These findings collectively suggest that the knocking down of *Lonp1* in MLE12 cells affected four aging-associated pathways, with the *Fgf2* gene potentially being a pivotal gene interconnecting them. To corroborate this, we assessed *Fgf2* mRNA expression in mouse lung tissue. Post-bleomycin tracheal instillation, the AT2 cell-specific *Lonp1* knockout mice showed an increase in *Fgf2* mRNA expression compared with the control group (*Sftpc*^CreERT2^*; Lonp1*^flox/+^) (Figure 6E). This was consistent with the trend of *Fgf2* changes in transcriptome sequencing. Furthermore, we validated the *Lonp1* mRNA expression levels in human lung tissue from IPF patients using data sourced from the GEO database. The analysis indicated that *Lonp1* mRNA levels were significantly reduced in the lung tissues of IPF patients when compared to healthy individuals (Figure 6F).

## Discussion

The *Lonp1* mutation was initially identified in the Cerebral, Ocular, Dental, Auricular, Skeletal (CODAS) syndrome [22]. Subsequent studies have shown that the *Lonp1* gene is associated with the occurrence and development of various diseases and conditions, including neurological disorders such as Parkinson’s disease [23], cardiovascular diseases [4, 24], neurodegenerative diseases [25], chronic kidney disease [26], skeletal muscle abnormalities [27], cancer, and mitochondrial diseases [10]. However, the role of *Lonp1* in the respiratory system has not been investigated. In patients with IPF, AT2 cells express aging markers, and the aging of AT2 cells was shown to promote IPF in a mouse model [12, 28]. Bleomycin damages the DNA of AT2 cells and activates p53 signaling, inducing the expression of SASP in AT2 cells [29]. The autocrine positive feedback of TGFβ in the AT2 cell lineage is central to the mechanisms of non-inflammatory pulmonary fibrosis, with the p53 and TGFβ signaling pathways collaboratively inducing the fibrotic transition from AT2 to AT1 cells [29]. Our study showed that knocking out the *Lonp1* gene in AT2 cells significantly reduced the number of AT2 cells, accompanied by a decrease in the number of AT1 cells. This phenomenon is likely to affect the self-repair ability of mouse alveolar epithelial cells stimulated by bleomycin. Furthermore, *Lonp1* knockout in AT2 cells increased the expression of aging markers, such as p53 and p21, in AT2 cells, indicating that knocking out the *Lonp1* gene promotes AT2 cells aging. One study suggested that TGF-β1, produced by fibroblasts, and its downstream factor interleukin (IL)-11 can promote peripheral AT2 cell aging and exacerbate pulmonary fibrosis [30]. Autocrine TGFβ in AT2 cells also promotes the transformation of AT2 cells into the profibrotic AT2 cells (PATS)/the damaged AT2 cells (DATS)/keratin 8 (KRT8) transition cells or the alveolar basal intermediates. The activation of TGFβ-related gene expression by p53 is crucial for triggering pulmonary fibrosis [29]. The inhibition of PAI-1 has been shown to block TGFβ-induced aging and the age-related secretory phenotype in AT2 cells [31]. PAI-1 also promotes p53 expression and activates the p53-p21-retinoblastoma protein (Rb) cell cycle inhibition pathway, ultimately leading to AT2 cell aging [32]. To determine whether the *Lonp1* gene deletion promoted the SASP in AT2 cells, we assessed the expression of the two aforementioned AT2-related SASP secretion factors. The results showed that the expression levels of TGF-β1 and PAI-1 were increased in mice with AT2-specific *Lonp1* gene knockout.

Initially, we intended to use primary AT2 cells to investigate how *Lonp1* gene deficiency induces aging in these cells. However, due to the insufficient extraction of primary AT2 cells, we opted for MLE12 cells, which are commonly utilized in IPF research. We performed transcriptome sequencing on MLE12 cells with knocked-out *Lonp1*. The sequencing data revealed an upregulation in the p53 signaling pathway and a downregulation in the cell cycle pathway following *Lonp1* knockdown, aligning with our in vivo findings.

The PI3K-AKT signaling pathway plays an important role in cell growth, proliferation, and metabolism [33]. Extensive research has shown that inhibiting the PI3K-AKT signaling pathway in fibroblasts may inhibit fibroblast activation and delay the pulmonary fibrosis progression [34–36]. Currently, several PI3K/AKT inhibitors, such as omipalisib (GSK2126458), HEC68498, and rapamycin, are being clinically evaluated in IPF patients [37–39]. Additionally, studies have shown that the activation of the insulin-like growth factor 1 (IGF1)/PI3K/AKT signaling pathway induces aging in AT2 cells [40]. Our findings demonstrated significant upregulation of the PI3K-AKT signaling pathway in MLE12 cells with *Lonp1* knockdown, suggesting a potential role for this pathway in the *Lonp1*-regulated aging of AT2 cells.

The NF-κB pathway is one of the important downstream signaling pathways of the PI3K-AKT signaling cascade. Activation of the NF-κB pathway induces cellular DNA damage and promotes aging in mice [41]. The inhibition of the NF-κB pathway reduced the expression of aging markers and SASP-related markers in AT2 cells following bleomycin stimulation [42]. Additionally, activation of this pathway has been found to facilitate the differentiation of lung resident mesenchymal stem cells into myofibroblasts [43]. Several recent studies have indicated that various drugs can attenuate bleomycin-induced pulmonary fibrosis by inhibiting the NF-κB pathway [44–47]. Our study found that knocking down *Lonp1* in MLE12 cells resulted in upregulation of the NF-κB pathway.

Based on the aforementioned findings, we hypothesize that the knockdown of *Lonp1* activates aging-related signaling pathways, induces AT2 cell aging, and exacerbates bleomycin-induced pulmonary fibrosis in mice. We used the GES53845 dataset from the GEO database to analyze the expression of the *Lonp1* gene in IPF patients. The analysis revealed that the expression level of *Lonp1* in the lung tissues of IPF patients was significantly lower compared to healthy individuals. However, due to the unavailability of relevant datasets, we could not analyze the expression of the *Lonp1* gene specifically in AT2 cells of IPF patients.

To elucidate the relationships among the aforementioned aging-related signaling pathways, we conducted a protein interaction network analysis, which identified *Fgf2* as a central hub gene connecting the four pathways. The expression level of the *Fgf2* gene is elevated in lung tissue of mice with bleomycin-induced and AT2 cell-specific *Lonp1* knockout. The *Fgf2* gene encodes the fibroblast growth factor 2 protein (FGF2), a member of the FGF family that is crucial for maintaining normal cell growth and repair [48]. FGF-2 is a potent mitogen for fibroblasts and is known to induce collagen synthesis in lung fibroblasts and myofibroblasts [49]. Additionally, TGF-β1 and reactive oxygen species (ROS) can induce overexpression of FGF-2 [50]. Activation of the FGF receptor (FGFR) by FGF ligands triggers several intracellular pathways, including the fibroblast growth factor receptor substrate 2 pathway, the extracellular signal-regulated kinases 1 and 2 (ERK1/2) pathway, the rat sarcoma virus (Ras)/rapidly accelerated fibrosarcoma (Raf)/mitogen-activated protein kinase (MAPK) pathway, and the PI3K-AKT pathway [51]. Epithelial cells in IPF patients exhibit overexpression of FGF-2 and FGFR-1 [52]. Our study also demonstrated that an increase in *Fgf2* gene expression correlates with upregulation of the PI3K-AKT pathway. Furthermore, other research has shown that FGF2 protein levels significantly increase in mice with pulmonary fibrosis induced by bleomycin. In a previous study, in vitro experiments revealed that FGF2 promotes the differentiation of lung mesenchymal cells into fibroblasts [53]. This study highlighted that blocking the wingless-related integration site (Wnt)/β-catenin signaling pathway may reduce FGF2 expression in both AT2 cell lines stimulated by bleomycin in vitro and in a mouse model of bleomycin-induced pulmonary fibrosis [53]. Another study showed that FGF2 stimulation significantly downregulated 29 genes in the Wnt signaling pathway and inhibited TGF-β-induced differentiation of lung pulmonary fibroblasts, as well as the expression of pro-fibrotic genes in these cells [54].

Our study elucidated the connection of the *Fgf2* gene within four aging-related pathways through transcriptome sequencing and demonstrated an increase in *Fgf2* gene expression in AT2 cell-specific *Lonp1* knockout mice following bleomycin treatment. This finding highlights a potential downstream target gene through which *Lonp1* may regulate AT2 cell aging. However, our research did not establish the specific pathway through which *Fgf2* promotes pulmonary fibrosis. Investigating the changes in *Fgf2*-related signaling pathways in IPF and understanding how the aging of AT2 cells, regulated by the *Lonp1* gene, contributes to fibrosis formation will be an important direction in our subsequent studies.

Our study demonstrated that the loss of the *Lonp1* gene promotes aging in AT2 cells; however, it did not determine whether this effect is mediated through changes in mitochondrial function. Previous studies have highlighted abnormal mitochondrial function in the AT2 cells of IPF patients, characterized by increased ROS generation, mtDNA damage, and reduced mitochondrial autophagy [55, 56]. Transcription factor 3 (ATF3) has been shown to disrupt mitochondrial homeostasis by inhibiting the synthesis of PTEN-induced putative kinase 1 (PINK1) mRNA. A conditional deficiency of ATF3 in AT2 cells protects mice from bleomycin-induced pulmonary fibrosis [57]. Additionally, deficiencies in the mitochondrial fusion proteins mitofusin 1 (MFN1) and mitofusin 2 (MFN2) can damage lipid metabolism in mouse AT2 cells, affecting surfactant lipid production and promoting the formation of spontaneous pulmonary fibrosis [58]. The results of this study have not yet been validated in lung tissue or AT2 cells of IPF patients. Moving forward, we plan to analyze the correlation between the *Lonp1* and *Fgf2* genes using lung tissue from IPF patients. Since FGF2 can be detected in plasma [59], we will also examine the relationship between serum FGF2 levels and clinical indicators to assess its potential role in the progression of IPF.

The ultimate goal of our efforts is to develop effective treatments for IPF. Our findings indicate that the absence of *Lonp1* exacerbates bleomycin-induced pulmonary fibrosis, yet it remains uncertain whether LONP1 activators could be utilized as a therapeutic strategy for IPF. One study highlighted an LONP1 activator, 84-B10, which has shown mitochondrial protective effects and antagonistic effects on renal fibrosis in mice [26]. Additionally, mitochondrial replication therapy (MRT), primarily based on the Pg-Fe-human mesenchymal stem cells (hMSC) strategy, has been effective not only in delivering mitochondria continuously and efficiently to lung epithelial cells but also in reversing the inhibition of mitochondrial autophagy. This therapy achieves the goal of repairing damaged lung epithelial cells and treating pulmonary fibrosis [60]. Furthermore, nitrated fatty acids (NFAs), a type of peroxisome proliferator-activated receptor γ (PPARγ) agonist, enhance PPARγ expression in fibroblasts and reduce TGFβ activity, exhibiting anti-fibrotic properties. NFA treatment not only impedes the pro-fibrotic effects of TGFβ but also facilitates the breakdown of collagen and reverses the transdifferentiation of myofibroblasts, ultimately ameliorating established pulmonary fibrosis in mice [61]. Several ongoing clinical trials offer promising prospects for the treatment of IPF. Results from a phase 2 clinical trial on phosphodiesterase 4 (PDE4) inhibition (BI1015550) showed that, regardless of whether patients received anti-fibrotic drugs, BI1015550 treatment can prevent pulmonary function decline in IPF patients within 12 weeks [62]. Additionally, engineered humanized bi-specific antibodies (SAR 156597), which block the IL-4 and IL-13 pathways, are currently being evaluated in phase 2 clinical trials [63].

## Conclusion

Our study has shown that the loss of the *Lonp1* gene exacerbates bleomycin-induced pulmonary fibrosis in mice. This effect may be attributed to the acceleration of AT2 cell aging resulting from *Lonp1* deficiency. The cell cycle pathway, p53 signaling pathway, PI3K-AKT signaling pathway, and NF-κB pathway appear to be involved in the *Lonp1*-mediated regulation of AT2 cell aging, with *Fgf2* emerging as a crucial gene connecting these pathways. Further research is required to elucidate the regulatory interactions between *Lonp1* and *Fgf2* and their associated downstream signaling pathways. Our findings introduce new gene targets for investigating the mechanisms underlying IPF.

## Data Availability

The datasets used and analyzed during the current study are available from the corresponding author upon reasonable request.
